# Peritubular and Tubulointerstitial Inflammation as Predictors of Impaired Viral Clearance in Polyomavirus Nephropathy

**DOI:** 10.3390/jcm13195714

**Published:** 2024-09-25

**Authors:** Haris Omić, Michael Eder, Tarek A. Schrag, Nicolas Kozakowski, Johannes Kläger, Gregor Bond, Željko Kikić

**Affiliations:** 1 Division of Nephrology and Dialysis, Department of Medicine III, Medical University of Vienna, 1090 Vienna, Austria; haris.omic@meduniwien.ac.at (H.O.); michael.eder@meduniwien.ac.at (M.E.); tarek.schrag@stpoelten.lknoe.at (T.A.S.); gregor.bond@meduniwien.ac.at (G.B.); 2 Department of Pathology, Medical University of Vienna, 1090 Vienna, Austria; nicolas.kozakowski@meduniwien.ac.at (N.K.);; 3 Department of Urology, Medical University of Vienna, 1090 Vienna, Austria

**Keywords:** kidney transplantation, polyomavirus, inflammation, peritubular capillaritis, PVN

## Abstract

**Introduction:** Polyomavirus-associated nephropathy (BKPyVAN) is a common complication in kidney transplant recipients. The histological changes in the context of BKPyVAN and their association with the viral load and outcomes are still being investigated. **Methods:** This retrospective study involved 100 adult patients transplanted between 2000 and 2021, with available archived biopsy slides, aiming to analyze associations between viral load clearance in the blood (reduction in BKPyVAN-DNAemia below detection level) and histological features in biopsy-proven BKPyVAN. A kidney pathologist blinded to the clinical data reassessed the BANFF 2019 lesion scores in the BKPyVAN index biopsy. The primary endpoint was viral clearance three months after the diagnosis. **Results:** The presence of tubulointerstitial inflammation, peritubular capillaritis, and higher PVN Class at the diagnosis was linked to a reduced likelihood of viral clearance three months later (interstitial inflammation OR = 0.2, 95% CI [0.07–0.55], tubulitis OR = 0.39, 95% CI [0.21–0.73], peritubular capillaritis OR = 0.25, 95% CI [0.08–0.82], PVN Score OR = 0.1, 95% CI [0.03–0.4]), independently of other covariates. Combining the four lesions using the ROC analysis enhanced their capability to predict persistent BK viremia after 3 months with an AUC of 0.94. **Conclusions:** The presence of interstitial inflammation, tubulitis, and peritubular capillaritis, as well as the higher PVN Score, was associated with an up to 90% lower likelihood of viral load clearance three months post-diagnosis. These findings underscore the importance of histological evaluation as a surrogate of subsequent viral clearance and offer valuable insights for the management of BKPyVAN.

## 1. Introduction

One of the most common viral infections in kidney transplant recipients is BK polyomavirus infection (BKV). It remains a clinically significant cause of progressive graft dysfunction, particularly in the first year after transplantation. The advanced disease also known as Polyomavirus nephropathy (BKPyVAN) causes a rapid progression of interstitial fibrosis, graft dysfunction, and reduced graft survival [1,2,3]. Timely detection of BKPyVAN typically leads to improved allograft survival compared to cases diagnosed at an advanced stage [4]. Despite several non-invasive methods to estimate disease risk, as well as the clinical course of a BKV infection, the histopathological examination of kidney biopsies remains one of the most important tools for diagnosing BKPyVAN and assessing its severity [5,6]. The histological hallmarks of BKPyVAN include viral cytopathic changes in tubular epithelial cells, interstitial inflammation, and fibrosis [7,8,9].

Persistent BK viremia is one of the most important risk factors for worse outcomes in BKPyVAN, while available clinical and histological parameters do not provide the level of accuracy needed to differentiate patients with a higher or lower likelihood of viral clearance [10]. Given the important role of allograft biopsy in facilitating early diagnosis and interventions in BKPyVAN, a deep understanding of the nuances of each histological feature becomes paramount [11,12]. The role of interstitial inflammation in BKPyVAN is still a subject of investigation, and its role as an indicator of lack of viral clearance in BKPyVAN cases remains to be thoroughly explored [13]. It has been shown that patients with a lower degree of inflammatory infiltrate display a lower viral load than patients with a higher degree of inflammatory infiltrate at the time of the BKPyVAN biopsy [14,15]. Summarized, tubulointerstitial inflammation has been described both in active BKPyVAN and resolving infection, underscoring the complexity of interpreting these histological features [13,16]. However, the correlation between the polyomavirus viral clearance and these histological changes has not yet been explored in this context. In the present study, our objective was to systematically assess the several histological lesions described in BKPyVAN by retrospective examination of the archived histological samples collected from a cohort of 100 patients over 20 years. Specifically, our evaluation encompassed the following aspects: (a) histological findings in the index biopsy; and (b) the correlation between these findings in BKPyVAN including PVN Class and its association with viral clearance three months after the biopsy [17,18,19,20] (Supplemental Materials Table S1).

## 2. Materials and Methods

### 2.1. Study Participants

All kidney transplant recipients who underwent allograft biopsies at the Medical University of Vienna from 2000 to 2021, and were histologically confirmed to have BKPyVAN, were considered eligible participants for this retrospective study. The inclusion criteria were individuals aged 18 years or older, a confirmed diagnosis of BKPyVAN in allograft biopsy using positive SV40-immunostaining, the availability of clinical and virological data with a 12-month follow-up period following the BKPyVAN diagnosis, and the availability of archived histology slides from the index biopsy. The primary endpoint of interest was the clearance of BK viral load in serum (defined as a reduction in viral load under the detection level; 70 copies/mL) observed three months after the histological diagnosis. The selection of this specific time frame was made with consideration of the existing literature, which varies in defining viral clearance at different time points, ranging from 12 weeks to several months. By choosing the three-month mark, we aimed to balance clinical relevance and consistency with prior research (reported median clearance time of 19.2 weeks; range 6.4–98.5 weeks and from 1 to 5 months [1,17]. Demographic and clinical parameters, including age, gender, date of kidney transplantation, underlying kidney disease, donor-specific factors, and treatment protocols, were retrospectively collected. The approach to managing acute graft rejection in this cohort was standardized according to our center’s usual practice [21].

### 2.2. BKPyVAN Screening

Per our facility’s protocol, all patients underwent BKPyVAN-PCR testing in serum after KTX. Initially, tests were conducted at every outpatient visit for the first three months, then monthly until the end of the first-year post-transplantation. Subsequently, the testing intervals were extended, typically occurring every three to six months, or more frequently if indicated. Standard of care for the treatment of BKPyVAN consisted of standardized reduction in CNI and/or MMF. After BKPyVAN diagnosis, the dose of MMF/Azathioprine was either reduced by half or discontinued. In a second step, the dose of the administered CNI was reduced (Tacrolimus levels were targeted to <6 ng/mL, CyA < 150 ng/mL). In rare cases, due to persistent viremia, experimental rescue treatments were used.

### 2.3. Histological Evaluation

The primary objective of this study was to conduct a detailed analysis of the impact of acute and chronic kidney allograft lesions on viral clearance in patients diagnosed with BKPyVAN following kidney transplantation. In all patients, the biopsies were carried out in response to increases in creatinine or proteinuria, accompanied by a positive BKPyVAN PCR test. These biopsies were evaluated on formalin-fixed paraffin-embedded sections applying the standard methodology. Biopsies were deemed suitable for the study if the preserved slides included at least 10 glomeruli and 2 arteries. The minimum acceptable sample was defined as containing 7 glomeruli and 1 artery from at least 2 distinct cores. To ensure adequate PVN-Class assessment, all specimens needed to have additional medulla in the biopsy. The 2019 BANFF histology scores were re-evaluated by one experienced kidney pathologist [22]. The pathologist was blinded to the patient’s characteristics and clinical outcome of interest. All BKPyVAN cases were tested for the presence of viral infection and the diagnosis of BKPyVAN was confirmed with immunohistochemical staining for SV40 large T antigen. The PVN Class was determined as the total percentage of SV40 large T antigen-positive tubular units in the biopsy specimen accompanied by fibrosis. The pvl (intrarenal polyomavirus load levels) score was obtained as reported previously (Supplemental Materials Table S1). Peritubular capillaritis was reassessed concerning its scope (focal: 10–50% of renal cortex PTC; diffuse: >50%), its severity (“ptc score”), and the composition of leukocytes involved (mononuclear, mixed, or neutrophilic) [23]. Lastly, we utilized the PVN classification provided by Nickeleit et al. to comprehensively evaluate BKPyVAN-related histological changes.

### 2.4. Statistical Analysis

The data obtained for this investigation were statistically and graphically analyzed using commercial software (Microsoft Office Pro Plus 365, Excel, Microsoft Corp., Redmond, WA, USA; IBM SPSS Statistics, Version 27.0, IBM, Armonk, NY, USA, and GraphPad Prism 10.0.3 Macintosh Version by Software MacKiev © 1994-2023 GraphPad Software, LLC, LAJ, USA). Continuous parameters were reported as mean (±standard deviation) if they met the criteria of a Gaussian distribution, otherwise reported with median and interquartile range (IQR). We employed binary logistic regression to model the probability of viral clearance after 3 months as the outcome variable, considering the histological parameters and their associated BANFF rejection scores as predictor variables. The histological lesions were evaluated in a binary fashion, anticipated due to the low count in each group, dividing them into two categories: positive or negative. Baseline variables that showed statistical differences or trends toward the difference between the groups were taken into account for potential inclusion in the multivariate model. Taking into account the long duration of our research, we examined the variations in outcomes and predictors between two different time periods, 2000–2010 and 2011–2021, in an attempt to lessen the possible effects of the diagnosis era. To exclude bias regarding the historical diagnosis of rejection and potential effects on the endpoint, we decided to include the historical biopsy diagnoses in our descriptive data. To evaluate the differences in viral load among different histology scores, we employed a one-way Analysis of Variance (ANOVA). To visualize the results of the binary logistic regression analysis, we employed a forest plot. The forest plot graphically presents the odds ratios (ORs) and their corresponding 95% confidence intervals (CIs). To evaluate the diagnostic accuracy and predictive power of these parameters for the outcome of viral clearance at three months, we conducted a Receiver Operating Characteristic (ROC) curve analysis. For the cross-validation analysis, we employed a three-fold cross-validation technique to assess the predictive performance of histological variables. AUC was calculated for each individual histological predictor and for two-way combinations of predictors, as well as for the combined model using all four predictors for each fold to ensure the robustness of the predictive model and to mitigate the risk of overfitting.

#### Guidelines

All relevant statements as per the STROBE guidelines have been implemented [24].

## 3. Results

### 3.1. Patient Cohort

The study included 100 adult patients presenting BKPyVAN in the biopsy between the years 2000 and 2021 (3598 KTX in the study period). The median age at the time of transplantation was 54 years (44.3–63.5), and sixty-seven (67%) patients were male. Seventy-nine (79%) received a deceased donor transplant (DD). Patient demographic and clinical data are displayed in Table 1. Eighty-five patients received their first kidney transplant (KTX), while 15 patients were retransplanted. All patients received standard immunosuppression (IS) with calcineurin inhibitor (CNI), antiproliferative agent, and steroids. Induction therapy with basiliximab was used in 31 (31%) of the patients while anti-thymocyte globulin (ATG) was used in 16 (16%) of the patients. Causes for end-stage renal disease (ESRD) are displayed in Supplemental Materials Table S2. The first positive BK virus PCR in blood was detected at a median of 113 (68.5–220) days after KTX. The BKPyVAN was diagnosed at the median of 181 (116–384) days. In all patients, the primary treatment for BKPyVAN during the first three months after diagnosis involved a reduction in IS medication. Viral clearance at month 3 was achieved by 22 patients (22%). The analysis of baseline clinical and demographic parameters between patients with viral clearance and viral persistence at 3 months revealed no differences. In the analysis of primary immunosuppression trough levels between the viral clearance and persistence groups, no significant differences were detected. Furthermore, the frequency of antimetabolite reduction was comparable between the groups. During the post-KTX surveillance period, a total of 139 kidney biopsies corresponding to 100 patients were performed before the index biopsy. The kidney allograft pathology observed in the pre-BKPyVAN biopsies is provided in detail in Supplemental Materials Table S3. The assessment of graft function, as determined by eGFR-CKD-EPI (mL/min/1.73 m^2^), initially revealed no notable distinctions between the two groups at the time of diagnosis. After six, nine, and twelve months following the diagnosis, notable alterations in graft function between the groups were evident, with CKD-EPI-eGFR of 41.0 ± 16.7 vs. 30.1 ± 18.2 (*p* = 0.02) at six months; Cohen’s d: −0.591 (95% CI [−1.103, −0.075]), 38.8 ± 16.1 vs. 28.9 ± 18.1 (*p* = 0.03) at nine months; Cohen’s d: −0.546 (95% CI [−1.070, −0.018]), and 38.5 ± 18.1 vs. 27.9 ± 18.8 (*p* = 0.04) at twelve months; Cohen’s d −0.585 (95% CI [−1.109, −0.058]), indicating a moderate difference between the two groups (Table 1).

### 3.2. Viral Load and Viral Load Clearance

**Figure 1:**
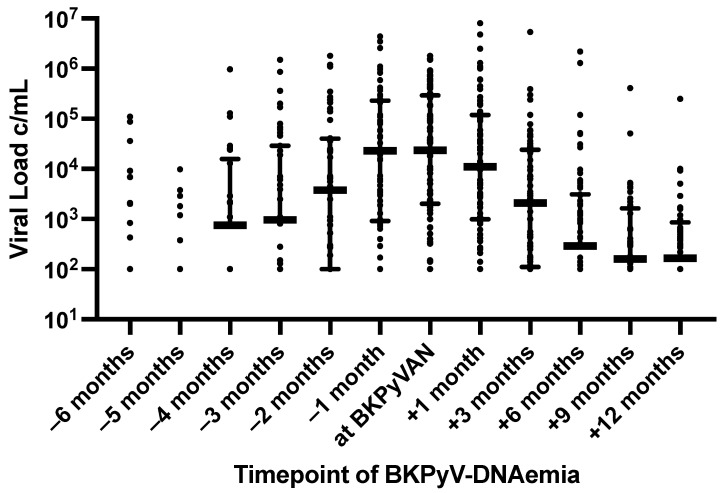
Aligned dot plot for the viral load before and after the Biopsy. Values displayed as Median/IQR. Zero values are not plotted due to the logarithmic axis. The medians for variables with a low number of cases (<10) were not plotted.

Viral load measurements were taken at various intervals following KTX. These measurements were performed monthly post-KTX on one hand, and during each patient visit following the diagnosis on the other hand. The complete viral clearance at month 3 as the primary outcome was attained by 22% of the patients in the study. The trajectory of viral load leading up to the diagnosis and after the biopsy for both the entire cohort and the respective groups is illustrated in Figure 1 and Supplemental Materials Figure S1, as well as in Supplemental Materials Table S4. It reached its highest level with a median of 2.3 × 10^4^ c/mL (9.2 × 10^2^–2.3 × 10^5^), one month preceding the biopsy, followed by stable values at the biopsy, and a consistent decline thereafter. The median viral load at the diagnosis of BKPyVAN was 2.3 × 10^4^ c/mL (2.1 × 10^3^–2.6 × 10^5^) and one month after the biopsy at 9.6 × 10^3^ c/mL (9.6 × 10^–1^ × 10^5^). The BKPyVAN-DNAemia at the diagnosis did not differ between the groups. Three months after the biopsy, the median viral load decreased further to 1 × 10^3^ c/mL (1 × 10^2^–1.8 × 10^4^). In the subsequent course, the viral load observed at months 6, 9, and 12 following the biopsy consistently decreased, with median viral load values of 1.4 × 10^2^ c/mL (0–3 × 10^3^), 120 c/mL (0–1.3 × 10^3^), and 165 c/mL (0–8.7 × 10^2^).

### 3.3. Histological Findings in BKPyVAN Biopsy

**Figure 2:**
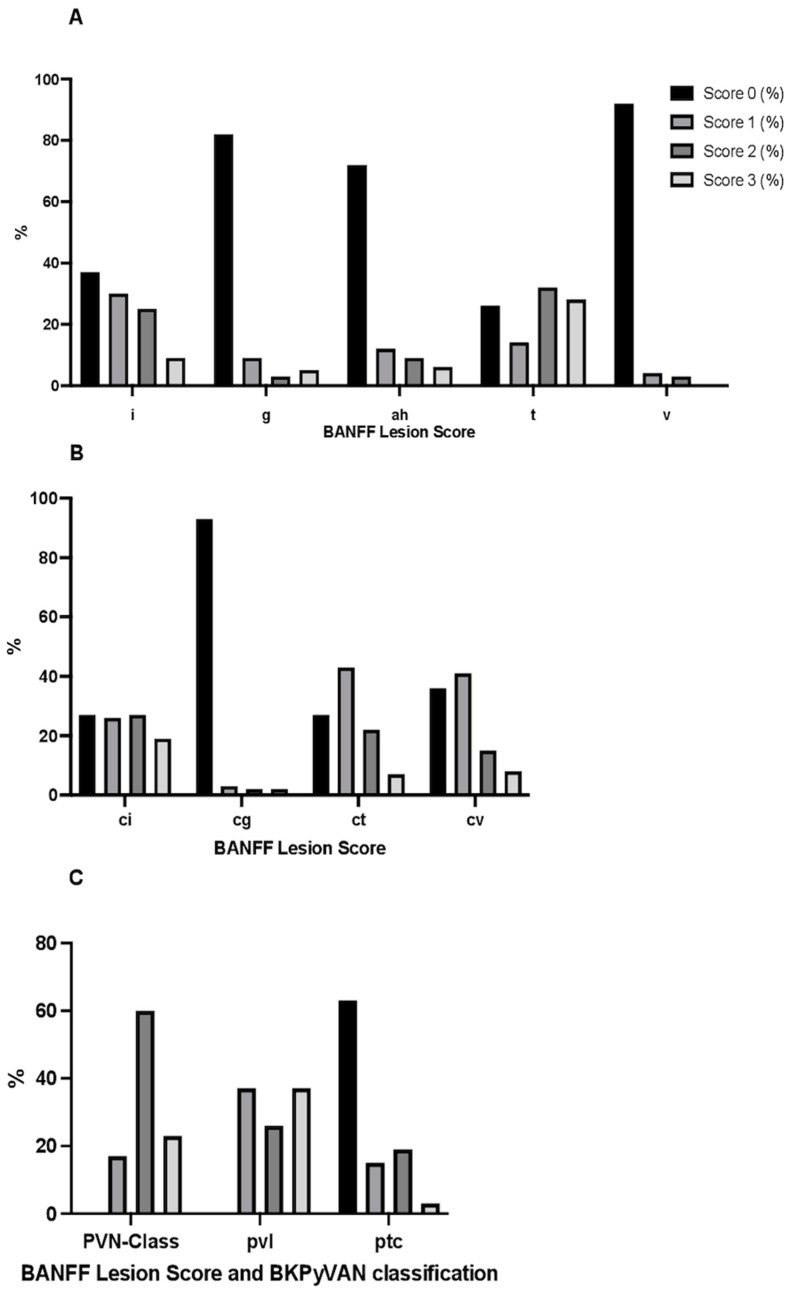
Stacked column graphs showing the distribution of BANFF lesion scores in the index biopsy. Panel design was used for easier visualization; g: glomerulitis, ah: arteriolar hyalinosis, t: tubulitis, v: intimal arteritis, cg: double contours, ci: interstitial fibrosis, ct: tubular atrophy, cv: intimal thickening, i: interstitial inflammation, ptc: peritubular capillaritis, PVN: PVN Class, pvl: intrarenal polyomavirus load levels.

The BKPyVAN was diagnosed at a median of 181 days after the KTX. The evaluation revealed a notable prevalence of specific BANFF lesions, providing further context to the histopathological landscape. Among them, interstitial inflammation (i) was evident in 62 (62%) cases. The most common was grade 1 with 29 (47%) cases. Interstitial fibrosis (ci), was present in 69 (69%) cases. Peritubular capillaritis (ptc) was evident in 37 cases, most commonly as grade 2 in 19 (51%) cases. The overall assessment as well as the exact occurrence of all lesions is displayed in Table 2 and Figure 2. Five lesions/classes were found to be significantly more prevalent in patients without viral clearance. As shown in Table 2, the presence of ptc, i, t, PVN Class, and a higher pvl score, a crucial part of PVN Classification, was significantly more frequently observed in these patients (PVN score phi = 0.448, *p* < 0.001, ptc phi = −0.307, *p* = 0.002, i phi = −0.324, *p* = 0.001, t phi = 0.306, *p* = 0.030, and pvl phi = 0.337, *p* = 0.003). Concomitant graft rejection, defined as the diagnosis of any rejection in the BKPyVAN biopsy, was identified in 14 cases (14%) within our study cohort. The tubulointerstitial inflammation was observed in 14 cases (14%). The most frequently observed rejection subtype was historically diagnosed as the T-cell-mediated rejection (TCMR) Grade 1A, which was noted in seven cases (50%). Additionally, ABMR was diagnosed in three (21%) patients where the glomerulitis was present.

### 3.4. BANFF Single Lesions and Virologic Findings

**Figure 3:**
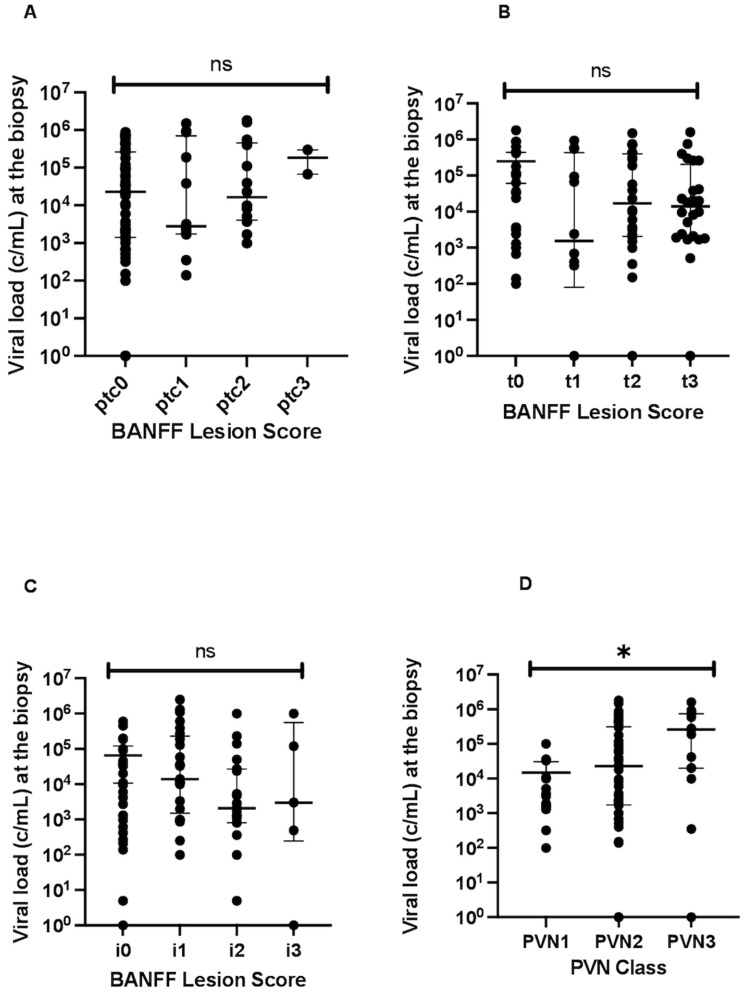
(**A**) Viral load at the BKPyVAN biopsy according to different peritubular capillaritis grades; *p* = 0.37, (**B**) viral load according to the interstitial inflammation grade; *p* = 0.87, (**C**) viral load according to the tubulitis; *p* = 0.58, (**D**) viral load according to the PVN Class; *p* = 0.001 (*). Values are displayed as median and IQR.

When examining the viral load at biopsy among patients with varying grades of peritubular capillaritis (ptc), we did not observe differences in viral load in patients with higher ptc scores (*p* = 0.37) depicted in Figure 3A. There was also no statistically significant difference for the assessment of interstitial inflammation (i) (*p* = 0.87; Figure 3B or tubulitis (t) (*p* = 0.58; Figure 3C. However, we observed that a higher PVN Class was significantly linked with an elevated viral load at the time of biopsy (*p* = 0.001) showing a direct linkage of viral load in plasma and kidney itself; Figure 3D. As mentioned earlier, we meticulously assessed both the presence and extent of peritubular capillaritis (ptc) while also considering the types of cells involved in these ptc lesions. Notably, in 19 (51.3%) of the cases, peritubular capillaritis manifested as focal lesions, covering 10–50% of the renal cortex. Additionally, in 18 (48.1%) of the biopsies with positive ptc findings, we identified diffuse changes associated with peritubular capillaritis. The composition of the leukocyte types involved in peritubular capillaritis revealed a striking consistency within our cohort. In nearly all cases (*n* = 35), the predominant leukocyte type involved was pure mononuclear.

### 3.5. Histological Parameters and Probability of Viral Load Clearance

**Figure 4:**
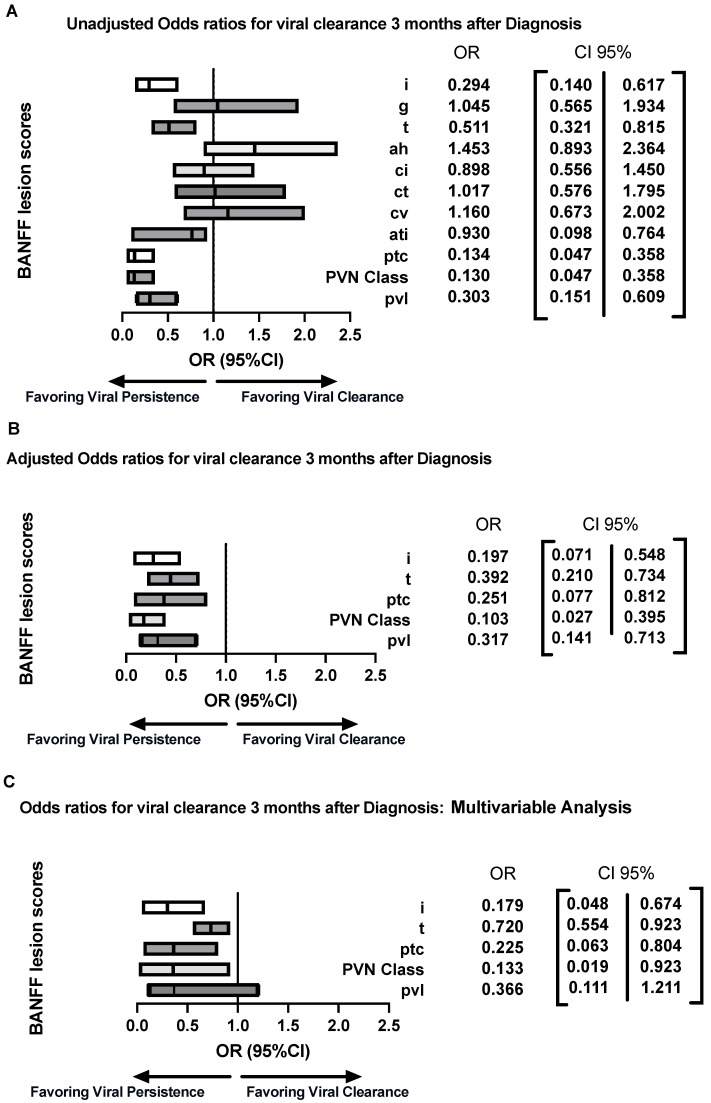
Forest plot displaying Odds ratios for viral load clearance in BKPyVAN three months after the diagnosis using a binary logistic regression model: (**A**) Unadjusted model; (**B**) model adjusted for viral load at the diagnosis (copies/mL); presence of any concomitant rejection and use of ATG induction therapy; (**C**) Multivariable analysis of histological lesions/PVN Classification for viral clearance after 3 months; i: interstitial inflammation, g: glomerulitis, t: tubulitis, ci: interstitial fibrosis, ct: tubular atrophy, cv: intimal thickening, ah: arteriolar hyalinosis, ati: acute tubulus injury, ptc: peritubular capillaritis, PVN: Polyoma viral load Class; pvl: intrarenal polyomavirus load levels. Values displayed as Odds Ratio for viral clearance three months after diagnosis of BKPyVAN (95% CI).

To evaluate the likelihood of predicting viral clearance three months post-treatment, we employed a binary logistic regression model. This model was utilized to analyze how the presence or absence of histological lesions affects the outcome. The logistic regression analysis yielded noteworthy results regarding the probability of viral clearance three months post-diagnosis. Specifically, the presence of tubulointerstitial inflammation was significantly associated with lower odds of viral clearance (interstitial inflammation (i) OR = 0.3, 95% CI [0.14–0.67], *p* < 0.01, tubulitis (t) (OR = 0.51, 95% CI [0.32–0.81], *p* < 0.01). Additionally, peritubular capillaritis was associated with reduced odds of viral clearance, with an OR of 0.27 (95% CI [0.1–0.76], *p* < 0.01). The PVN Class also showed a significant association, with an OR of 0.13 (95% CI [0.04–0.35], *p* < 0.01); Figure 4A. The pvl score, a critical component of the PVN Classification, was also significantly associated with viral clearance, demonstrating an OR of 0.30, with a 95% (CI) of 0.14–0.71, *p* < 0.01. Despite no differences in the baseline variables between the two groups, to enhance the robustness of our findings, we subsequently analyzed all significant lesions for potential confounding factors within our study population, including viremia at the time of diagnosis, the use of anti-thymocyte globulin (ATG), and the presence of any concomitant rejection. After adjustments for these covariates, we found that our previously significant results remained consistent (Figure 4B). In the multivariable analysis of lesion scores, four variables demonstrated significant associations with viral clearance at three months: PVN score OR = 0.13, 95% CI [0.02–0.92], *p* = 0.041, peritubular capillaritis OR = 0.23, 95% CI [0.06–0.80], *p* = 0.022, interstitial inflammation OR = 0.18, 95% CI [0.05–0.67], *p* = 0.011, and tubulitis (OR = 0.720, 95% CI [0.55–0.92], *p* = 0.05 (Figure 4C). The pvl score did not show a significant association with the endpoint in the multivariable analysis, suggesting that the PVN score itself carries more predictive information regarding the outcome in BKPyVAN. The analysis of the frequency of BANFF lesion scores across two predefined timeframes, 2000–2010 and 2011–2021, revealed no statistically significant differences in the distribution of relevant lesion scores (*p* > 0.05 for all variables, Table 3).

### 3.6. Histological Parameters and Effects on Graft Function after 12 Months of Follow-Up

We conducted a series of linear regression analyses to investigate how histological lesions affect or predict clinical outcomes, specifically serum creatinine levels 12 months after diagnosis. The predictors included were those previously associated with viral load and other important clinical covariates. For peritubular capillaritis the reported coefficient (B) was 0.552 (95% CI: 0.001 to 1.103, *p* = 0.050), indicating that each unit increase in severity of peritubular capillaritis is associated with 0.552 higher SCr levels after 12 months. The tubulitis showed a coefficient (B) of 0.243 (95% CI: −0.149 to 0.635, *p* = 0.219), suggesting no significant impact. For interstitial inflammation, the coefficient (B) was 0.643 (95% CI: 0.167 to 1.119, *p* = 0.009), indicating substantial effects on higher SCr. The PVN-Class also showed a significant correlation, with a coefficient (B) of 0.836 (95% CI: 0.099 to 1.572, *p* = 0.027).

### 3.7. Histological Parameters and Delta Serum Creatinine after 12 Months of Follow-Up

To further explore the impact of histological lesions on kidney function, we conducted linear regression analyses to investigate the association between histological parameters and changes in SCr levels 12 months post-biopsy (Delta SCr). For interstitial inflammation, the coefficient (B) was 0.737 (95% CI: 0.419 to 1.055, *p* < 0.001), indicating that each unit increase in the severity of interstitial inflammation is associated with a 0.737 unit increase in SCr levels after 12 months. Peritubular capillaritis showed a coefficient (B) of 0.476 (95% CI: 0.091 to 0.860, *p* = 0.016), indicating that each unit increase in the severity of peritubular capillaritis is associated with a 0.476 unit increase in SCr levels after 12 months. The PVN-Score had a coefficient (B) of 0.577 (95% CI: 0.021 to 1.133, *p* = 0.042), suggesting that each unit increase in the PVN-Score is associated with a 0.577 unit increase in SCr levels after 12 months. Tubulitis had a coefficient (B) of 0.368 (95% CI: 0.057 to 0.679, *p* = 0.021), indicating that each unit increase in the severity of tubulitis is associated with a 0.368 unit increase in SCr levels after 12 months.

### 3.8. Sub-Analysis of Graft Function in Patients without Viral Clearance after Three Months

To evaluate graft function in patients without viral clearance, we performed a sub-analysis of the patients who did not achieve viral clearance three months post-biopsy by analyzing the viral reduction and graft function during the follow-up. The median viral load reduction in this cohort was −2.3 × 10^4^ copies/mL (IQR −8 × 10^2^ to −2.7 × 10^5^ copies/mL). To investigate the impact of viral load reduction on kidney function during the follow-up period, we conducted a Pearson correlation analysis. The results demonstrated a significant positive correlation between the delta of viral load at three months and creatinine levels at six months post-BKPyVAN (rho = 0.512, *p* < 0.001), nine months post-BKPyVAN (rho = 0.476, *p* < 0.001), and twelve months post-BKPyVAN (rho = 0.304, *p* = 0.028). These findings suggest that a greater reduction in viral load is associated with improved kidney function throughout the follow-up period even if the complete clearance has not been achieved.

### 3.9. Risk Prediction and ROC Analysis

**Figure 5:**
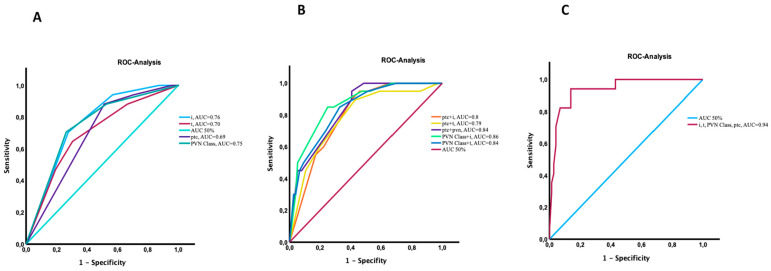
Receiver Operating Characteristic (ROC) analyses for assessment of the diagnostic accuracy of four crucial histological parameters for viral load persistence at month 3—interstitial inflammation (i), PVN-Class, peritubular capillaritis (ptc), and tubulitis (t): (**A**) Interstitial inflammation: AUC = 0.76, PVN-Class: AUC = 0.75, Peritubular capillaritis: AUC = 0.69 Tubulitis: AUC = 0.70; (**B**); AUC results for combinations of each histologic parameters; (**C**) AUC for integrated analysis = 0.94.

In the study, we used Receiver Operating Characteristic (ROC) analyses to assess the value of four crucial histological parameters from the univariable analysis—interstitial inflammation, PVN Class, peritubular capillaritis, and tubulitis—in predicting the lack of viral clearance at the three-month mark post-diagnosis in patients with BKPyVAN. Individual ROC analyses were performed for each parameter to determine their respective capacities in discriminating between patients who experienced viral load persistence and those who achieved viral clearance by the end of the third month. The results yielded the following area under the curve (AUC) values for interstitial inflammation 0.76, PVN Class = 0.75, peritubular capillaritis = 0.69, and tubulitis = 0.70. Subsequently, to further investigate the combined impact of these parameters, we conducted integrated ROC curve analyses for each of the two distinct combinations: tubulitis, inflammation, PVN Class, and ptc. The obtained area under the curve (AUC) values and associated statistics for these combinations are presented in Figure 5B. The combination of the four risk factors enhanced its predictive capability, amalgamating all four parameters and providing a comprehensive assessment of their collective strength in predicting the lack of viral clearance. The AUC value for this composite analysis was 0.94 (Figure 5C).

#### Cross-Validation of ROC Analysis

In the cross-validation analysis, we assessed the predictive performance of different histological variables in determining viral clearance at 3 months, using three-fold cross-validation for robustness. The area under the ROC curve (AUC) was calculated for individual predictors, two-way combinations of predictors, and for the model combining all four predictors. The AUC for individual predictors, including PVN Class, ptc, interstitial inflammation, and tubulitis, ranged from 0.67 to 0.78, with PVN showing the highest AUC at 0.78. For two-way combinations, the AUCs ranged from 0.64 to 0.82, with the combination of PVN and i having the highest AUC at 0.82. The combined model incorporating all four predictors yielded an AUC of 0.80, demonstrating that combining multiple histological features improves the model’s predictive power in our cross-analysis model. Although the AUCs in the cross-validation analysis were slightly lower compared to the original analysis (AUC for combined analysis in original model 0.94; in Cross-Validation 0.80), they remained within acceptable ranges, demonstrating the robustness of the predictive model across different folds. This analysis confirms that even with slight variability, the predictive capacity of the model remains strong (Supplemental Materials Table S5).

## 4. Discussion

The present study examined BKPyVAN in kidney transplant recipients comprehensively, with a particular focus on histological changes and their influence on viral clearance. It provided insights into the intricate relationship between histological parameters, viral load dynamics, and clinical outcomes. We assessed Banff lesions indicating acute injury, with a particular emphasis on interstitial inflammation, and their impact on viral clearance in patients with BKPyVAN. We investigated whether the severity of interstitial inflammation played a significant role in the ability to clear the viral load. Further on, we described and analyzed the effect of tubulitis (t), and sought to examine how the extent of tubulitis impacted the clearance of viral load, considering its potential role as a marker of missing viral clearance describing active BKPyVAN infection. As a novelty in BKPyVAN, peritubular capillaritis (ptc), characterized by inflammation in the peritubular capillaries, was re-assessed to understand its potential as a surrogate for viral clearance. The analysis of histological parameters, including interstitial inflammation (i), tubulitis (t), peritubular capillaritis (ptc), and PVN Class (PVN), revealed noteworthy associations with viral clearance. The presence of these lesions was found to decrease the likelihood of viral clearance by as much as 90%, irrespective of other clinically important covariates such as rejection, induction therapy with ATG, and the peak viral load at the biopsy. While these results underscore the potential of a multifaceted approach in enhancing predictive accuracy, they should be interpreted cautiously, considering the overlapping information these related lesions might share. We observed significant differences in kidney function over a 12-month period among patients, depending on their viral clearance three months after diagnosis. This suggests that early viral clearance and its assessment could be of crucial importance for this patient group. Our findings align closely with one of the pivotal studies in the field of BKPyVAN conducted by Drachenberg and colleagues [15]. Their study highlighted a significant association between active inflammation at the time of diagnosis and worse graft outcomes in BKPyVAN. Building on these crucial insights, our study employed a model to assess the likelihood of lower viral clearance as one of the most important variables influencing graft function [17]. This relationship became particularly evident when examining graft function post-diagnosis, as patients who achieved early viral clearance exhibited stable graft function compared to those without viral clearance, who demonstrated a notable decrease in eGFR. Notably, we found that interstitial inflammation provided similar results, mirroring the findings in the study by Drachenberg and his team. Similarly, Masutani and colleagues made a comparable observation, albeit in a slightly different context [25]. They observed that patients with elevated tubulointerstitial inflammation experienced a significant rise in creatinine levels. While some of this increase was reversible, it never fully reverted to the initial baseline. This finding emphasizes the potential scenario wherein increased inflammation may signify a prolonged infection, eventually leading to irreversible alterations [25]. The work by Menter and colleagues on the pathology of “Resolving” BKPyVAN provides important insights into the dynamics of inflammation and viral load in this condition [23]. Their observation of increasing tubulointerstitial inflammation as the polyoma viral load decreases is a noteworthy finding. It suggests that, in some cases, inflammation may persist even after complete viral clearance. Our findings align with this study where persistent inflammation could indeed be indicative of an ongoing resolution of the polyomavirus, which however may have prolonged complete clearance or lead to less effective viral elimination. In this context, we analyzed the impact of previously described histological lesions on kidney allograft function, measured by serum creatinine levels 12 months after diagnosis as well as the effects of these lesions on SCr changes. Peritubular capillaritis was associated with a significant rise in serum creatinine, indicating that the severity of this lesion could also predict the worsening of the kidney allograft function (B = 0.552, 95% CI: 0.001 to 1.103, *p* = 0.050).

Conversely, tubulitis showed no significant effect on long-term renal function (B = 0.243, *p* = 0.219), suggesting possible resolution without lasting damage. Interstitial inflammation notably affected serum creatinine levels (B = 0.643, *p* = 0.009), underscoring its contribution to the progression of chronic renal damage through its role in fibrosis development [26]. Furthermore, the PVN-Class, reflecting polyomavirus-associated nephropathy, significantly correlated with increased serum creatinine (B = 0.836, *p* = 0.027), highlighting the clinical importance of assessing viral damage early. Similar findings have been seen for the changes in serum creatinine 12 months after diagnosis, further supporting our findings.

These parallel findings strengthen the role of interstitial/tubulointerstitial inflammation in BKPyVAN and emphasize its potential utility as a predictive factor for viral clearance. Ptc, often indicative of kidney rejection, suggests an ongoing immune response and inflammation within the renal tissue. The presence of ptc in BKPyVAN was also documented in a study conducted by Xu-Tao and colleagues. Among the 19 biopsies they examined, 11 of them exhibited the presence of ptc [27]. This finding aligns with our observations, stressing the consistency of this histological feature in BKPyVAN across different research studies. Such consistency reinforces the significance of ptc as an important prognostic criterion in BKPyVAN, showing that the presence of ptc reduces the likelihood of viral clearance by up to 75%. The PVN Classes provided by Nickeleit et al. proved to be an important tool for assessing BKPyVAN-related histological changes and their association with viral clearance [17,19,20]. Providing the PVN Class in the BKPyVAN histology proved once again to be an important finding, being associated with the prolonged disease. In previous works, the assessment of a higher PVN Class indicated significantly worse clinical outcomes. Our results corroborate these observations, demonstrating a significantly reduced probability of viral clearance—up to 90%—in cases presenting with elevated PVN Class scores. Given that the pvl score did not display an association in the multivariable analysis, it can be inferred that the PVN classification, which integrates both pvl and fibrosis, predominantly dictates the risk of viral persistence. This supports the use of PVN classification as the current classifier in BKPyVAN. The findings reported by Drachenberg and colleagues in 2017 highlighted an intriguing aspect of BKPyVAN management. According to their study, successful viral clearance could not be reliably predicted using either histological or clinical data. Instead, they observed that higher Log BK viremia at the time of the first and second biopsies was associated with a reduced likelihood of viral clearance [13]. Interestingly, our data, while partially contrasting with their findings, also partially align with them. With our larger sample size, we were able to demonstrate that certain lesion scores predict the patients at a lower likelihood of achieving viral clearance. To support these findings, we carried out individual ROC analyses for four pivotal histological parameters—interstitial inflammation, PVN Class, peritubular capillaritis, and tubulitis—to discern their abilities in predicting viral persistence at the three-month mark in patients with BKPyVAN. The results of these individual analyses revealed area under the curve (AUC) values of approximately 0.7 for each parameter, signifying their moderate predictive capabilities when assessed in isolation. However, when we unified these four parameters into a comprehensive composite analysis, a remarkable transformation in predictive accuracy was observed. The combined ROC analysis yielded a substantially higher AUC value of 0.94. Such a robust collective assessment can significantly contribute to the development of more accurate prognostic tools for BKPyVAN, providing a new important surrogate for the course of viral load.

In our cross-validation analysis, while the AUC values were slightly lower compared to the initial analysis, the model still demonstrated good predictive performance. Despite no significant differences in viral load at the time of biopsy, the presence of these histological features was associated with challenges in clearing the virus. This underscores the multifactorial nature of BKPyVAN, where the outcome results from a complex interplay among different factors, including viral load, immune response, and tissue damage [28,29,30]. To avoid the confounding effects that may arise from anti-rejection therapies on viral clearance, we specifically adjusted our results for this variable among others. The presence of rejection, which was treated according to a standardized protocol, did not affect viral clearance in our cohort. Key histological features, such as peritubular capillaritis, interstitial inflammation, and the PVN score, remained significantly associated with viral persistence. This highlights the robustness of our results and emphasizes that histological markers, rather than therapeutic interventions alone, may be important drivers of viral outcomes in our cohort. Further on, we assessed the levels of primary immunosuppression between the viral clearance and persistence groups, and no significant differences were observed. Additionally, the frequency of antimetabolite reduction did not differ between the groups, further supporting the robustness of our findings. While this consistency in immunosuppressive regimens across groups strengthens the validity of our results, it is important to acknowledge that variability in medication adherence and individual patient responses to immunosuppressive therapy can introduce subtle deviations that may not be fully captured in the analysis. These factors, although standardized within our study design, represent potential limitations and are inherent to clinical practice. Regarding the results of our study and the previous literature, it is to be assumed that the viral load, histological parameters, and the immune response all contribute to the overall outcome [31,32,33,34]. Our study delivers valuable findings, but its retrospective methodology over 20 years has flaws that may contribute to selection bias and data discrepancies, as well as the possibility that historical diagnosis of concomitant rejection may have impacted the endpoints. One of the limitations of the study is the possibility that an anti-BK-specific immune response, potentially triggered by the reduction in immunosuppression, could have influenced the morphological phenotype observed at the time of biopsy. However, given that the histological diagnosis typically coincided with peak viremia and that the primary intervention during this period was limited to immunosuppression reduction, we believe that the observed viral dynamics during these three months were primarily reflective of the natural course of the infection and the initial therapeutic response after the biopsy. Nonetheless, these concerns were addressed through strict selection criteria, centralized biopsy evaluation, and adjustment of relevant results for these covariates. Another potential limitation of our study is that it only includes patients who underwent a transplant biopsy for confirmation of BKPyVAN. Consequently, our findings cannot be directly applicable to patients who are monitored clinically without biopsy. It is important to acknowledge that these patients may experience different disease courses, as the decision to biopsy may be influenced by clinical symptoms, viral load, or other risk factors. The findings from our study suggest that the existence of inflammation in any section of the biopsy should be approached with caution, as it may signal an extended period for viral clearance. This observation underscores the need for more closely monitored follow-ups in such cases. While our study identified significant associations between histological features, viral load clearance, and graft function, the importance of these findings highlights the need for validation in future studies utilizing independent cohorts in a multicenter setting to further establish their robustness and generalizability.

## Figures and Tables

**Table 1:** Basic clinical and demographic characteristics of the study population.

Variables	Total N = 100	Viral Clearance after 3 Months N = 22	Viral Persistence after 3 Months N = 78	*p*-Value
**Baseline**				
Male N (%)	67 (67%)	17 (77.3%)	50 (64.1%)	0.31
Age at transplantation (y) Median (IQR)	54.0 (44.3–63.5)	58.5 (46.7–64.2)	51.5 (44–62.5)	0.39
Donor age (y) Median (IQR)	55.0 (46.0–66.5)	57.0 (30.0–72.0)	55.0 (46.0–66.0)	0.82
First KTX N (%)	85 (85%)	18 (81.8%)	67 (85.9%)	0.73
>1 KTX N (%)	15 (15%)	4 (18.2%)	11 (14.1%)	n.d.
DD N (%)	79 (79%)	15 (68.2%)	64 (84.2%)	n.d.
MM sum; Median (IQR)	3 (2–4)	3 (2–4)	3 (1.5–4)	0.21
Cold ischemia time (h) Median (IQR)	14 (5.3–18.0)	12 (3–16)	14.5 (8–19)	0.20
DSA at the KTX N (%)	8 (8%)	1 (6.7%)	7 (11.7%)	0.52
Induction Therapy: ATG, N (%)	16 (16%)	1 (4.5%)	15 (23.4%)	0.1
Any rejection before Diagnosis N (%)	11 (11%)	3 (13%)	8 (10%)	0.80
**At Diagnosis**				
Time KTX to first positive BK-PCR (d) Median (IQR)	113 (68.5–220)	113 (61.5–146)	116 (69.5–248)	0.43
Time KTX to BKPyVAN (d) Median (IQR)	181 (116–384)	190 (113.5–411.2)	181 (119–381.5)	0.90
eGFR CKD-EPI at the BKPyVAN Diagnosis mL/min/1.73 m^2^ Mean (SD)	34.8 (14.3)	35.9 (13.4)	34.6 (16.9)	0.72
Any concomitant rejection in the index biopsy N (%)	14 (14%)	4 (18%)	10 (12%)	0.50
BKPyVAN-DNAemia at the diagnosis, c/mL	2.3 × 10^4^ (2.1 × 10^3^–2.6 × 10^5^)	1.4 × 10^4^ (2.1 × 10^3^–9.7 × 10^4^)	3.5 × 10^4^ (2.1 × 10^3^–3.1 × 10^5^)	0.32
Tacrolimus-Based-Immunosuppression N (%)	92 (92%)	19 (86.3%)	65 (90.2%)	0.68
Tacrolimus trough level at Diagnosis Mean (SD)	7.04 (2.58)	6.89 (2.39)	7.19 (2.75)	0.39
Tacrolimus trough level at Month 1 Mean (SD)	6.81 (2.83)	6.71 (2.87)	6.92 (2.79)	0.40
Tacrolimus trough level at Month 3 Mean (SD)	6.44 (2.40)	6.88 (2.75)	6.01 (1.99)	0.19
Reduction in Antimetabolites N (%)	48 (48%)	10 (45%)	38 (52%)	0.50
**During Follow-Up**				
eGFR 6 months after BKPyVAN Mean (SD)	32.7 (18.3)	41.0 (16.7)	30.1 (18.2)	0.02
eGFR 9 months after BKPyVAN Mean (SD)	31.0 (18.6)	38.8 (16.1)	28.9 (18.1)	0.03
eGFR 12 months after BKPyVAN Mean (SD)	30.1 (18.4)	38.5 (18.1)	27.9 (18.8)	0.04
Δ eGFR Index Biopsy-Month 12 Mean (SD)	−4.67 (13.5)	+0.94 (11.9)	−6.11 (13.6)	0.04
Δ eGFR Month 3–Month 12 Mean (SD)	−3.95 (11.9)	+1.23 (9.5)	−5.4 (12.6)	0.03
Time of Diagnosis 2000–2010, N (%)	48 (48%)	10 (20.8)	38 (79.2)	0.79
Time of Diagnosis 2011–2021, N (%)	52 (52%)	12 (23.1)	40 (76.9)	

Abbreviations: KTX: Kidney transplantation, DD: deceased donor, MM: mismatch, DSA: Donor specific antibodies, ATG: anti-thymocyte globulin BK PCR: BK virus Polymerase Chain Reaction, BKPyVAN: BK Polyomavirus Nephropathy.

**Table 2:** Distribution of BANFF lesion scores in the Index Biopsy.

Lesions	Score	Viral Clearance after 3 Months N = 22	Viral Persistence after 3 Months N = 78	Total	*p*-Value
i	0	14 (66.7%)	22 (28.6%)	36	0.004
	≥1	7 (33.3%)	55 (71.4%)	62	
PVN-Class	1	10 (45.5%)	7 (9.0%)	17	<0.001
	≥2	12 (54.5%)	71 (90.9%)	83	
g	0	17 (81.0%)	62 (82.7%)	79	0.970
	≥1	4 (19.0%)	13 (17.3%)	17	
ah	0	12 (57.1%)	58 (75.3%)	70	0.390
	≥1	9 (42.9%)	18 (24.7%)	27	
t	0	10 (50.0%)	15 (19.7%)	25	0.030
	≥1	10 (50.0%)	61 (80.3%)	71	
v	0	17 (85.0%)	66 (94.3%)	83	0.342
	≥1	3 (15.0%)	4 (5.7%)	7	
cg	0	18 (90.0%)	70 (92.1%)	88	0.496
	≥1	2 (10.0%)	6 (7.9%)	8	
ci	0	5 (27.8%)	21 (27.3%)	26	0.875
	≥1	13 (72.2%)	56 (72.7%)	69	
ct	0	6 (31.6%)	20 (26.3%)	26	0.872
	≥1	13 (68.4%)	56 (73.7%)	69	
cv	0	4 (21.1%)	27 (35.5%)	31	0.315
	≥1	15 (78.9%)	49 (64.5%)	64	
ptc	0	20 (90.9%)	43 (55.1%)	63	0.023
	≥1	2 (9.1%)	35 (44.9%)	37	
pvl	1	15 (68.2%)	22 (28.2%)	37	<0.001
	2	5 (22.7%)	21 (26.9%)	26	
	3	2 (9.1%)	35 (44.0%)	37	

I: Interstitial inflammation, PVN-Class: Polyoma viral load Classification, g: glomerulitis, ah: Arteriolar hyalinosis, t: tubulitis, v: intimal arteritis, cg: Double contours, ci: Interstitial fibrosis, ct: Tubular atrophy, cv: Intimal thickening, ptc: peritubular capillaritis, pvl: intrarenal polyomavirus load levels.

**Table 3:** Comparative Distribution of relevant BANFF Lesion Scores Across Two Time Periods (2000–2010 vs. 2011–2021).

Lesions	Score	2000–2011	2012–2023	Total	*p*-Value
i	0	20 (42.6%)	16 (31.4%)	36	0.377
	1	14 (29.8%)	15 (29.4%)	29	
	2	8 (17.0%)	16 (31.4%)	24	
	3	5 (10.6%)	4 (7.8%)	9	
PVN-Class	1	7 (14.6%)	10 (19.2%)	17	0.813
	2	30 (62.5%)	30 (57.7%)	60	
	3	11 (22.9%)	12 (23.1%)	23	
t	0	9 (19.1%)	16 (32.7%)	25	0.401
	1	8 (17.0%)	5 (10.2%)	13	
	2	17 (36.2%)	14 (28.6%)	31	
	3	13 (27.7%)	14 (28.6%)	27	
ptc	0	29 (60.4%)	34 (65.4%)	63	0.649
	1	9 (18.8%)	6 (11.5%)	15	
	2	8 (16.7%)	11 (21.2%)	19	
	3	2 (4.2%)	1 (1.9%)	3	

Abbreviations: I: Interstitial inflammation, PVN-Class: Polyoma viral load Classification, t: tubulitis, ptc: peritubular capillaritis.

## Data Availability

The raw data supporting the conclusions of this article will be made available by the authors upon request.

## Supplementary Materials

The following supporting information can be downloaded at: https://www.mdpi.com/article/10.3390/jcm13195714/s1: Supplemental Materials Table S1: Definition of PVN-Classes. Supplemental Materials Table S2. The underlying condition of the study population. Supplemental Materials Table S3. Key histologic findings in pre-BKPyVAN biopsies. Supplemental Materials Table S4. Dynamics of BK-Plasma Viral Load Relative to BKPyVAN Diagnosis at Various Time Points. Supplemental Materials Table S5: Cross-Validation of AUC for Viral persistence 3 months after Diagnosis. Supplemental Materials Figure S1. Aligned dot plot for the viral load before and after the Biopsy.
