# Supplementary material for: Peritubular and Tubulointerstitial Inflammation as Predictors of Impaired Viral Clearance in Polyomavirus Nephropathy
Source: J Clin Med. 2024 Sep 25;13(19):5714. doi: 10.3390/jcm13195714 (PMC11476510; doi:10.3390/jcm13195714)

Supplemental Table S1: Definition of PVN Classes

PVN-Score	pvl	ci
**PVN Class 1**	1	≤ 1
**PVN Class 2**	1	≥ 2
	2	0 – 3
	3	≤ 1
**PVN Class 3**	3	≥ 2

ci, Banff interstitial fibrosis score; pvl, intrarenal polyomavirus load levels.
Polyomavirus load – pvl 1: ≤ 1% of all tubules/ducts with viral replication, pvl 2: >1% to ≤ 10% of all tubules/ducts with viral replication, pvl 3: > 10% of all tubules/ducts with viral replication.
Banff interstitial fibrosis score – ci0: interstitial fibrosis in up to 5% of cortical area, ci1: mild, interstitial fibrosis in 6 to 25% of cortical area, ci2: moderate, interstitial fibrosis in 26 to 50% of cortical area, ci3: severe, interstitial fibrosis in more than 50% of cortical area.

Supplemental Table S2. The underlying condition of the study population

Cause of end-stage renal disease	Total *N = 100*	Viral persistence after 3 Months N=78	Viral clearance after 3 Months N=22	P Value
Diabetic nephropathy	9 (9 %)	7 (8.9%)	2 (9.1%)	n.s
Goodpasture Syndrom	2 (2 %)	2 (2.6%)	0 (0.0%)	n.s
Hypertensiv nephropathy	3 (3 %)	2 (2.6%)	1 (4.5%)	n.s
ADPKD	15 (15 %)	12 (15.4%)	3 (13.6%)	n.s
Kidney atrophy	15 (15 %)	12 (15.4%)	3 (13.6%)	n.s
Unknown	18 (18 %)	15 (19.2%)	3 (13.6%)	n.s
FSGS	2 (2 %)	2 (2.6%)	0 (0.0%)	n.s
Chronic infection	2 (2 %)	1 (1.3%)	1 (4.5%)	n.s
Glomerulonephritis	8 (8 %)	7 (9.0%)	1 (4.5%)	n.s
IgA Nephropathy	9 (9 %)	6 (7.6%)	3 (13.6%)	n.s
Acute kidney injury	2 (2 %)	2 (2.6%)	0 (0.0%)	n.s
Vascular nephropathy	3 (3 %)	1 (1.3%)	2 (9.1%)	n.s
Other	10 (10 %)	7 (9.0%)	3 (13.6%)	n.s

Supplemental Table S3. Key histologic findings in pre-BKPyVAN biopsies

**Time to first biopsy after KTX (BX1) Median (IQR) (d)**	6 (0-24)
**Time to second biopsy after KTX (BX2) Median (IQR)(d)**	27 (5-103)
Total number of biopsies N prior to BKPyVAN	139
Any rejection N (%)	12 (8.6%)
Borderline changes N (%)	11 (8%)
1a TCMR N (%)	1 (0.7%)
2a TCMR N (%)	4 (2.8%)
2b TCMR N (%)	2 (1.4%)
ABMR N (%)	5 (3.5%)

KTX: Kidney transplantation, BX: biopsy, TCRM: T-cell mediated rejection, d: Days, IQR: inter-quartile range, ABMR: Antibody-mediated rejection

Supplemental Table S4. Dynamics of BK-Plasma Viral Load Relative to BKPyVAN Diagnosis at Various Time Points

Time Relative to BKPyVAN Diagnosis	*Viral Clearance after 3 Months*	*Viral Persistence after 3 Months*	*P-value*
– 6 months	1.0 x 10^2 (0 - 2.025 x 10^3)	0 (0 - 7.38 x 10^2)	0.628
– 5 months	1.0 x 10^2 (0- 1.235 x 10^3)	0 (0 - 4.5 x 10^2)	0.467
– 4 months	4.6 x 10^2 (0 - 1.135 x 10^3)	1.0 x 10^2 (0 - 1.3 x 10^4)	0.692
– 3 months	5.45 x 10^2 (0 - 1.675 x 10^4)	2.25 x 10^2 (0 - 6.775 x 10^3)	0.945
– 2 months	9.3 x 10^2 (3.43 x 10^2 - 3.1025 x 10^4)	3.8 x 10^3 (1.0 x 10^2 - 2.2 x 10^4)	0.659
– 1 month	3.4 x 10^3 (1.7 x 10^2 - 4.2 x 10^5)	1.5 x 10^4 (1.15 x 10^3 - 7.0 x 10^4)	0.248
At BKPyVAN	1.40 x 10^4 (2.1 x 10^3 - 9.775 x 10^4)	3.5 x 10^4 (2.025 x 10^3 - 3.15 x 10^5)	0.322
+1 month	7.4 x 10^2 (1.825 x 10^2 - 1.25 x 10^4)	1.1 x 10^4 (1.6 x 10^3 - 1.05 x 10^5)	0.024
+3 months	0	3.5 x 10^3 (3.0 x 10^2 - 3.0 x 10^4)	<0.001
+6 months	0 (0 - 7.5 x 10^1)	1.0 x 10^3 (1.0 x 10^2 - 4.4 x 10^3)	0.031
+9 months	0 (0 - 1.075 x 10^2)	3.3 x 10^2 (0 - 2.075 x 10^3)	0.102
+12 months	0 (0 - 1.6 x 10^2)	1.35 x 10^2 (0 - 6.025 x 10^2)	0.102

Temporal dynamics of the BK-Plasma Viral Load across several intervals in relation to the BKPyVAN diagnosis. Values displayed as median (IQR). Statistically significant variations were noted at 3 months ($p < 0.001$), 6 months ($p < 0.001$), and 9 months ($p = 0.031$) post-diagnosis.

Supplemental Table S5. Cross-Validation of AUC for Viral persistence 3 months after Diagnosis

Predictors	AUC Fold 1	AUC Fold 2	AUC Fold 3	Mean AUC
PVN class	0.81	0.76	0.78	0.78
ptc	0.65	0.73	0.63	0.67
i	0.7	0.8	0.7	0.73
t	0.76	0.74	0.51	0.67
PVN class + ptc	0.81	0.88	0.77	0.82
PVN class + i	0.86	0.74	0.86	0.82
PVN class + t	0.9	0.73	0.73	0.79
ptc + i	0.72	0.86	0.63	0.76
ptc + t	0.71	0.86	0.63	0.73
PVN + ptc + i + t	0.84	0.81	0.74	0.8

Supplemental Figure S1. Aligned dot plot for the viral load before and after the Biopsy.

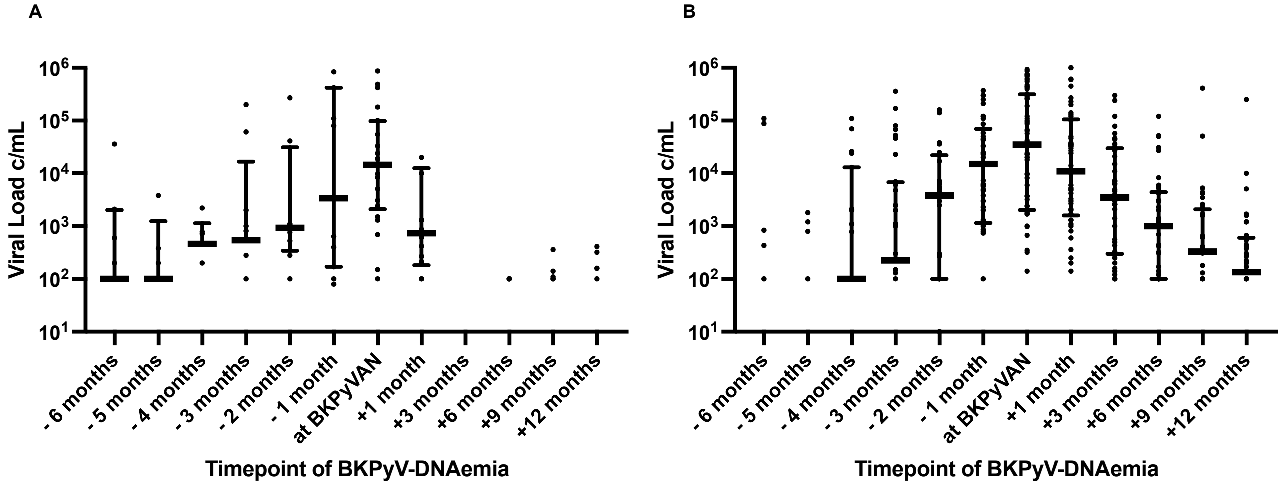

Supplement: Supplementary file 1 [file jcm-13-05714-s001.zip › jcm-3205597-supplementary.pdf]
